# Impact of DNA extraction techniques and sequencing approaches on microbial community profiling accuracy

**DOI:** 10.3389/frmbi.2025.1688681

**Published:** 2025-12-16

**Authors:** Polina Zoruk, Maxim Morozov, Vladimir Veselovsky, Aleksandra Strokach, Vladislav Babenko, Ksenia Klimina

**Affiliations:** Lopukhin Federal Research and Clinical Center of Physical-Chemical Medicine of Federal Medical Biological Agency, Moscow, Russia

**Keywords:** 16S rRNA gene amplicon sequencing, DNA extraction methods, illumina sequencing, metagenomic sequencing, microbiota profiling, Oxford Nanopore Technologies, ZymoBIOMICS gut microbiome standard

## Abstract

**Background:**

Quality control in metagenomic data analysis is crucial for ensuring the accuracy and reliability of research results. Among the key steps in microbiome research, DNA extraction plays a critical role, as it directly determines DNA yield, integrity, and representation of microbial taxa.

**Results:**

We compared three commercial DNA extraction kits and our protocol specifically developed for the recovery of high molecular weight (HMW) DNA from complex microbial communities, using the ZymoBIOMICS Gut Microbiome Standard. The PureLin^™^ Microbiome DNA Purification Kit and our custom protocol provided superior recovery of DNA from Gram-positive bacteria, while the Wizard^®^ kit and our protocol yielded HMW DNA suitable for long-read Oxford Nanopore sequencing. Among sequencing approaches, metagenomic sequencing on the Illumina platform provided the most accurate representation of the reference composition. However, all methods showed limited ability to detect taxa below 0.5% of relative abundance. Additionally, taxonomic classification based on 16S rRNA gene amplicon sequencing data misclassified closely related species due to high gene homology, a limitation not observed with metagenomic approaches.

**Conclusions:**

Our study establishes that a customized DNA extraction protocol is optimal for comprehensive microbiome studies utilizing long-read sequencing technologies. We show that metagenomic sequencing outperforms 16S rRNA gene amplicon sequencing for species-level accuracy, providing a validated benchmark for future gut microbiome research.

## Background

The study of microbiota has attracted increasing attention due to its profound implications in both fundamental and applied sciences. Microbial communities play crucial roles in human health, agriculture, and industrial biotechnology. Metagenomic analysis, which enables comprehensive identification and functional profiling of microbial communities, is a key tool for advancing public health, sustainable environmental practices, and biotechnological innovation (Gu et al., 2019; Taş et al., 2021).

Among the essential steps in sequencing, DNA extraction directly impacts genomic coverage and taxonomic resolution. Inefficient lysis - particularly of Gram-positive bacteria – or DNA fragmentation can substantially alter the apparent community composition (Karstens et al., 2021; Kazantseva et al., 2021; Rajar et al., 2022; Wright et al., 2023; Lourenço et al., 2024; Pu et al., 2025). Recognizing this, initiatives such as the MicroBiome Quality Control (MBQC) project and the International Human Microbiome Standards (IHMS) emphasize the importance of reliable DNA extraction for reproducible outcomes (Costea et al., 2017; Leigh Greathouse et al., 2019; Nouws et al., 2020; Nielsen et al., 2022).

Commercial DNA extraction kits vary in performance, each with specific advantages and limitations. Protocols such as MagicPure^®^ Stool and Soil Genomic DNA Kit and PureLink^™^ Microbiome DNA Purification Kit rely on mechanical lysis, which can effectively disrupt resilient cells but often results in fragmented DNA that is suboptimal for long-read sequencing (Thomas et al., 2012; Yuan et al., 2012). In contrast, the Wizard^®^ Genomic DNA Purification Kit excludes mechanical disruption and enables the recovery of high molecular weight (HMW) DNA, which is essential for Oxford Nanopore Technologies (ONT) and other long-read sequencing technologies.

16S rRNA gene amplicon sequencing remains a widely used approach for microbiota profiling. On the Illumina platform, sequencing of short regions such as V3–V4 or V4–V5 of 16S rRNA gene (~450 bp) provides high read accuracy (> 99%) but limited resolution, generally at the genus level (Ranjan et al., 2016; Larin et al., 2024). By contrast, ONT enables sequencing of the full-length 16S rRNA gene amplicon (V1–V9), allowing for species-level identification (Cha et al., 2023). Nevertheless, amplicon-based methods are less precise in characterizing microbial diversity then whole metagenome sequencing (MS) (Khachatryan et al., 2020). Comprehensive metagenomic analysis allows not only the identification of microorganisms in a sample but also the characterization of their potential biological and ecological roles, provided that sufficient genome coverage is achieved.

The ZymoBIOMICS Gut Microbiome Standard has become a widely used benchmark for evaluating DNA extraction methods, sequencing platforms, and bioinformatics pipelines. This synthetic microbial community mimics the diversity of gut microbiota and can help in evaluating consistency and reproducibility across studies (Mori et al., 2023; Gulyás et al., 2024; Isokääntä et al., 2024). In this study, we compared three commonly used DNA extraction kits — MagicPure^®^ Stool and Soil Genomic DNA Kit (TransGen Biotech, China), Wizard^®^ Genomic DNA Purification Kit (Promega, USA), and PureLink^™^ Microbiome DNA Purification Kit (Thermo Fisher Scientific, USA) — alongside our protocol specifically developed for the extraction of HMW DNA from fecal microbiota, suitable for subsequent sequencing on platforms such as ONT. Our aim was to assess how different DNA extraction techniques influence the composition and quality of microbiota profiles obtained through sequencing.

## Methods

### DNA extraction

In this study, we used ZymoBIOMICS Gut Microbiome Standard (D6331, Zymo Research), a mixture of cells from pure cultures of 18 bacterial strains, 2 fungal strains, and 1 archaeal strain in staggered abundances to simulate a typical gut microbiome. Nucleic acids were extracted using three commercial kits – PureLink™ Microbiome DNA Purification Kit (Thermo Fisher Scientific, USA), MagicPure^®^ Stool and Soil Genomic DNA Kit (TransGen Biotech, China), and Wizard^®^ Genomic DNA Purification Kit (Promega, USA) – and a custom protocol. For clarity, the following abbreviations are used throughout the text:

- ZymoMS_Thermo – PureLink™ Microbiome DNA Purification Kit (Thermo Fisher Scientific, USA).
- ZymoMS_Promega – Wizard^®^ Genomic DNA Purification Kit (Promega, USA).
- ZymoMS_KingFisher – MagicPure^®^ Stool and Soil Genomic DNA Kit (TransGen Biotech, China).
- ZymoMS_Modified – Custom protocol.

### PureLink™ microbiome DNA purification kit

DNA extraction from ZymoBIOMICS Gut Microbiome Standard was performed according to our previous protocol (Strokach et al., 2025). Briefly, 75 µL of the sample was supplemented with 400 µL of phosphate-buffered saline (PBS), transferred to bead tubes and homogenized (30 s 7,000 x g) using MagNA Lyser (Roche, Switzerland). The homogenate was centrifuged for 1 min at 7,000 × g (Dlab D3024, Dlab Scientific co. ltd., Beijing, China), and the resulting supernatant was used for DNA extraction following the manufacturer’s instructions.

### MagicPure^®^ stool and soil genomic DNA kit

The sample (75 µL) was mixed with 400 µL of PBS and transferred to MagNA Lyser Green Beads (Roche, Switzerland) bead tubes for homogenization on a MagNA Lyser (30 s 7,000 x g) (Roche, Switzerland). After homogenization, the sample was centrifuged for 1 min at 7,000 × g (Dlab D3024, Dlab Scientific co. ltd., Beijing, China). The supernatant was transferred to a new tube, 20 µL of proteinase K was added, and the mixture was incubated for 20 min at 65°C. DNA extraction was then performed according to the manufacturer’s protocol for the MagicPure Stool and Soil Genomic DNA Kit (TransGen Biotech, China) using a KingFisher™ Purification System (Thermo Fisher Scientific, USA).

### Wizard^®^ genomic DNA purification kit

To 75 µL of the sample, 480 µL of 50 mM EDTA and 120 µL of lysozyme (10 ng/µL) were added. The sample was incubating at 37°C for 60 min, that centrifuged for 2 min at 20,000 × g (Dlab D3024, Dlab Scientific co. ltd., Beijing, China), and the supernatant was discarded. DNA was extracted from the resulting pellet according to the manufacturer’s instructions for the Wizard^®^ Genomic DNA Purification Kit (Promega, USA).

### Custom protocol

DNA extraction was performed as described previously (Strokach et al., 2025). The sample (75 µL) was transferred to a 15 mL tube, and 2 ml of 50 mM EDTA along with 500 µL of 20 mg/mL lysozyme (Sigma-Aldrich, USA) was added. The sample was incubated at a thermal shaker (Allsheng, China) at 37°C 65 x g for 1.5 h with continuous mixing. After incubation, the sample was centrifuged at 3,000 x g for 2 min using a Centrifuge 5804 R (Eppendorf, Germany), and the supernatant was transferred to a new tube. The remaining pellet was resuspended in 1 ml of Nuclei Lysis Solution (Wizard^®^ Genomic DNA Purification Kit), homogenized by pipetting, and incubated in a thermal shaker (Allsheng, China) at 56°C and 300 x g for 1.5 h.

Following incubation 400 µL of Protein Precipitation Solution (Wizard^®^ Genomic DNA Purification Kit) was added, vortexed briefly (1 s), and incubated on ice for 10 min. The mixture was centrifuged at 3,700 x g for 10 min (Dlab D3024, Dlab Scientific co. ltd., Beijing, China), and the supernatant was transferred to a new 15 mL tube containing 1.4 mL of isopropanol. After incubation at −20°C for 1 h, the sample was centrifuged at 3,700 × g for 20 min (Centrifuge 5804 R, Eppendorf, Germany), and the DNA pellet was washed twice with 80% ethanol, air-dried for 10–15 min, and resuspended in 200 µL of Low TE buffer (10 mM Tris-HCl, pH 8.0; 0.1 mM EDTA).

For additional purification, an equal volume of 2% CTAB solution (100 mM Tris pH 8.0, 20 mM EDTA, 1.4 M NaCl, pre-warmed to 55 – 65°C) was added to the DNA, mixed gently, and incubated at 65°C for 10 min. An equal volume of chloroform was then added, vortexed for 30 s, and centrifuged at 3,500 x g for 5 min (Dlab D3024, Dlab Scientific co. ltd., Beijing, China). The upper aqueous phase was transferred to a new tube, and mixed with 1.1 volumes of 1% CTAB solution (50 mM Tris pH 8.0, 10 mM EDTA, pre-warmed to 55–65°C), and centrifuged at 3,500 x g for 5 min (Dlab D3024, Dlab Scientific co. ltd., Beijing, China), resulting in visible DNA precipitation. The pellet was dissolved in 0.5 mL of 10 mM Tris pH 8.0, 0.1 mM EDTA, and 1 M NaCl by heating at 65°C for 30 min, followed by the addition of 0.6 volumes of isopropanol. The mixture was gently inverted and centrifuged at 3,500 x g for 8 min (Dlab D3024, Dlab Scientific co. ltd., Beijing, China). The DNA pellet was washed twice with 0.5 mL of 70% ethanol, air-dried, and resuspended in 100 µL of Low TE buffer, followed by incubation at 65°C for 30 min to ensure complete solubilization.

DNA extracted with each protocol was quantified using a Qubit 4 Fluorometer and a Nanodrop ND-1000 spectrophotometer (Thermo Fisher Scientific, USA).

### 16S rRNA gene amplicon sequencing on the Illumina platform

For amplification of extracted DNA (1–5 ng), 16S rRNA gene amplicon primers targeting the V3-V4 region and incorporating 5’-Illumina adapter sequences (Forward Primer: 5’ TCGTCGGCAGCGTCAGATGTGTATAAGAGACAGCCTACGGGNGGCWGCAG; Reverse Primer: 5’ GTCTCGTGGGCTCGGAGATGTGTATAAGAGACAGGACTACHVGGGTATCTAATCC) were used (Evrogen Russia). PCR amplification was performed using the Tersus Plus PCR kit (Evrogen, Russia) in a total volume of 25 µL. Library preparation and sequencing on the Illumina platform were carried out as described previously (Larin et al., 2024). The libraries were sequenced on a MiSeq instrument (Illumina, USA) using the 500-cycle MiSeq reagent kit v2 (Illumina, USA) with 20% PhiX spike-in.

### 16S rRNA gene amplicon sequencing on the MinION platform

PCR amplification, library preparation, and sequencing on the MinION platform were performed according to our previous protocol (Strokach et al., 2025). Briefly, the full-length 16S rRNA gene amplicon was amplified using primers 27F (AGAGTTTGATYMTGGCTCAG) and 1492R (GGTTACCTTGTTAYGACTT). PCR amplification was performed using the Tersus Plus PCR kit (Eurogen, Russia) in a total reaction volume of 25 μL. The cycling conditions were as follows: initial denaturation at 95°C for 2min, (95°C for 1 min, 60°C for 1 min, and 72°C for 3 min), 27 cycles, followed by a final extension at 72°C for 2 min and 4°C – cooling. Amplicon quality was verified by electrophoresis in 1.5% agarose gel. The PCR products were purified using KAPA HyperPure Beads (Roche, Switzerland) according to the manufacturer’s protocol.

For sequencing the prepared library (12 µL) was mixed with 37.5 µL of sequencing buffer and 25.5 µL of loading beads, loaded onto an R10.4.1 flow cell (FLO-MIN114; Oxford Nanopore Technologies), and sequenced using the MinION Mk1B device. Data acquisition was performed using MINKNOW software v24.06.14.

### Metagenome sequencing on the Illumina platform

Extracted DNA (100 ng) was used for library preparation with the KAPA HyperPlus Kit (Roche, Switzerland) following the manufacturer’s instructions. Library underwent a final cleanup using KAPA HyperPure Beads (Roche, Switzerland), after which the library size distribution and quality were assessed using a High Sensitivity DNA chip (Agilent Technologies). Quantification was performed using the Quant-iT DNA Assay Kit, High Sensitivity (Thermo Fisher Scientific, USA). Sequencing was performed on the NextSeq 1000 (Illumina, USA) using the NextSeq 1000/2000 P2 Reagents kit (200 cycles) v3, with 2% PhiX (Illumina) added as an internal control.

### Metagenome sequencing on the PromethION platform

HMW DNA (1 μg) was used for library preparation according to the manufacturer’s protocol (Oxford Nanopore Technologies, UK). Sequencing libraries were prepared using the Ligation sequencing kit (SQK-LSK114-XL), native barcoding expansion kit (SQK-NBD114.96) and run on an R10.4.1 (FLO-PRO114M) flow cell using a PromethION device. Long reads were generated and basecalled using Dorado v.7.6.7 using default parameters (high accuracy (HAC) model, minimum quality value > 7).

### Bioinformatics analysis of 16S rRNA gene amplicon data from ONT and Illumina platforms

Reads obtained from ONT sequencing were processed using Porechop v0.2.4 (https://github.com/rrwick/Porechop) to remove adapters and PCR primers, with default parameter settings. Reads with Phred quality score below 10 and those shorter than 1,300 nucleotides (nt) or longer than 1,800 nt were filtered out using Chopper v0.6.0 (De Coster and Rademakers, 2023) with the settings -l 1300 and –maxlength 1800. Taxonomic classification was performed with the Emu pipeline v3.4.5 (Curry et al., 2022) with default parameters. Read quality statistics were generated using NanoStat v1.6.0 (De Coster and Rademakers, 2023). The processed data were then imported into RStudio (version 2024.04.2 + 764, R 4.4.1) for downstream analysis using the MicrobiotaProcess package v1.16.1 (Xu et al., 2023).

Raw reads obtained from Illumina sequencing were filtered and trimmed using Fastp v0.23.4 (Chen et al., 2018), followed by further processing with the DADA2 pipeline v1.26.0 (Callahan et al., 2016) was used to carry out further processing. Taxonomic annotation was carried out using the SILVA database v138.1 (Quast et al., 2013). The MicrobiotaProcess package v1.16.1 (Xu et al., 2023) was then used to evaluate the collected data.

To enable direct comparison, quality-filtered ONT reads and merged Illumina paired-end reads were aligned against a reference database containing the reference genomes of the organisms included in the ZymoBIOMICS Gut Microbiome Standard (https://s3.amazonaws.com/zymo-files/BioPool/D6331.refseq.zip). Alignment was performed using BLAST v2.16.0 with the following parameters: e-value of 0.00001, minimum query coverage per high-scoring pair of 70%, and a maximum of one target sequence per query (-max_target_seqs 1).

### Bioinformatics analysis of metagenomic data from Illumina and ONT platforms

ONT reads were preprocessed using Porechop v0.2.4 (https://github.com/rrwick/Porechop) with default parameter settings and Chopper v0.6.0 (De Coster and Rademakers, 2023) for filtering reads with Phred score below 10. To remove host (human) sequences, reads were aligned to the human reference genome GRCh38 (https://www.ncbi.nlm.nih.gov/datasets/genome/GCF_000001405.40) using Minimap2 v2.26 (Li, 2018). Taxonomic classification of the remaining reads was performed using the Emu pipeline v3.4.5 (Curry et al., 2022) with default parameters. Filtered reads were also mapped to the ZymoBIOMICS Gut Microbiome Standard reference (https://s3.amazonaws.com/zymo-files/BioPool/D6331.refseq.zip) using the BWA software package v0.7.18 (Li, 2013) with its default settings, and mapping statistics were calculated using SAMtools v1.21 (Danecek et al., 2021).

Illumina metagenomic reads were quality-checked with FastQC (https://www.bioinformatics.babraham.ac.uk/projects/fastqc/). Low-quality sequences and adapters were removed from the raw data using Fastp v0.23.4. Human reads were filtered out by aligning against the GRCh38 genome using HiSAT2 v2.2.1 (Kim et al., 2019). Taxonomic profiling was performed with MetaPhlAn4 v4.0.6 (Blanco-Míguez et al., 2023). The MicrobiotaProcess package v1.16.1 (Xu et al., 2023) was used to analyze the collected data.

Additionally, quality-filtered reads were aligned to the ZymoBIOMICS Gut Microbiome Standard reference database comprising the reference genomes of the organisms included in the Standard (https://s3.amazonaws.com/zymo-files/BioPool/D6331.refseq.zip). Alignment was performed using BLAST v2.16.0 with the following parameters: e-value of 0.00001, minimum query coverage per high-scoring pair of 70%, and a maximum of one target sequence per query (-max_target_seqs 1). Alignments with > 95% identity were retained, and average percent identity per reference species was calculated.

## Results

### Comparison of the efficiency of DNA extraction methods

Three commercial kits and a custom protocol were evaluated based on several key parameters, including total processing time, cell wall disruption method, DNA quantification, final DNA yield, purity and optical density (**Table 1**). The ZymoMS_Modified protocol had the longest processing time (400 min), as the extended duration of certain steps promotes the extraction of HMW DNA. The highest DNA concentration was obtained using the ZymoMS_KingFisher protocol (2.67 ng/μL), while the other methods yielded comparable concentrations ranging from 1.1 to 1.3 ng/μL. DNA purity was assessed using A260/A280 and A260/A230 spectrophotometric ratios. ZymoMS_Promega and ZymoMS_Modified showed no evidence of contamination, with A260/A280 values of 1.98 and 2.01, and A260/A230 values of 1.86 and 1.85, respectively. Notably, ZymoMS_Modified demonstrated slightly higher DNA purity compared to ZymoMS_Promega. All samples were evaluated by electrophoresis; however, those extracted using ZymoMS_Thermo and ZymoMS_KingFisher lacked HMW DNA and exhibited pronounced fragmentation. Consequently, these samples were not subjected to spectrophotometric analysis and were excluded from ONT sequencing. For the further sequencing, all DNA samples were pooled in equimolar amounts to ensure comparable read depths across samples (**Supplementary Table S1**).

**Table 1:** Comparison of DNA extraction methods from three commercial kits and custom protocol.

Extraction kits	Protocol duration (min)	Cell wall disruption method	DNA concentration method	Sample concentration, ng/μL	DNA quality	A260/A280	A260/A230	N50 (Illumina/ONT)	Mean Q-value (Illumina/ONT)
ZymoMS_Thermo	55	Mechanical	Silica Spin Column	1.1	Fragmented	Was not measured	Was not measured	101/Was not measured	33/Was not measured
ZymoMS_KingFisher	50	Chemical	Magnetic beads	2.67	Fragmented	Was not measured	Was not measured	101/Was not measured	33/Was not measured
ZymoMS_Promega	160	Chemical	Isopropanol precipitation	1.17	High- molecular	1.98	1.86	101/1,368	33/11
ZymoMS_Modified	400	Mechanical	Isopropanol precipitation	1.3	High- molecular	2.01	1.85	101/10,142	33/13

For every sequencing technique, alpha diversity metrics, including the Shannon and Simpson indices, were calculated (**Supplementary Table S2**). The Shannon diversity of the V3–V4 region (Illumina) and full-length 16S rRNA gene (ONT) showed a statistically significant difference as determined by the Kruskal-Wallis test (p = 0.0163). However, no significant difference was observed between MS on the Illumina and ONT platform (p = 0.4561). Importantly, no significant differences in Shannon indices were detected when comparing variations within each sequencing technique across different DNA extraction protocols. Similarly, Simpson diversity index showed no significant differences between sequencing techniques or among extraction protocols. These findings suggest that, although the observed diversity may be influenced by the choice of sequencing technology, alpha diversity in this artificial community is not affected by the choice of extraction protocol within a given sequencing strategy.

### 16S rRNA gene amplicon sequencing of the V3–V4 region on the Illumina platform

**Figure 1:**
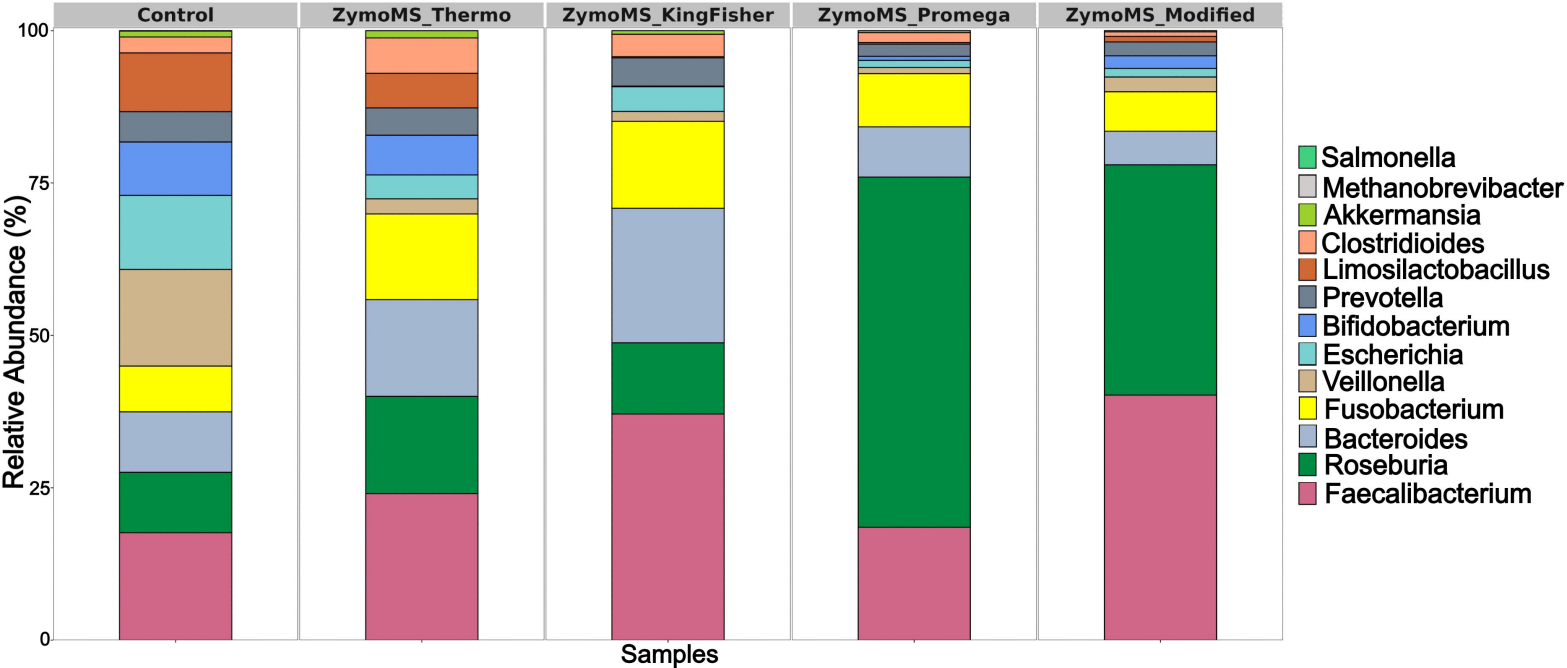
Relative abundances of bacterial genera identified using the V3–V4 region of the 16S rRNA gene amplicon sequencing on the Illumina platform. Bar plots show the relative abundances (%) of bacterial genera detected across four DNA extraction methods in comparison with the theoretical composition of the ZymoBIOMICS Gut Microbiome Standard (Сontrol). All detected taxa are shown; however, those with relative abundances below 0.5% are difficult to distinguish due to their low representation. Sequencing of the V3–V4 region on the Illumina platform enables reliable genus-level profiling, confirming the presence of major bacterial taxa included in the standard. Detailed abundance data are provided in **Supplementary Table S3**.

The sequencing data obtained for the V3–V4 region of the 16S rRNA gene amplicon using the Illumina platform are summarized in **Supplementary Table S1**. Sequencing of this region provided reliable genus-level profiles. All bacterial genera present in the ZymoBIOMICS Gut Microbiome Standard at an abundance above 0.5% were successfully identified (**Figure 1**). In contrast, taxa with relative abundances below 0.5% — *Methanobrevibacter smithii, Salmonella enterica, Enterococcus faecalis*, and *Clostridium perfringens* — were below the reliable detection threshold of the DADA2 pipeline and were not consistently detected across all replicates (**Supplementary Table S3**).

The presence of these low-abundance species was confirmed by BLAST alignment of reads against reference genomes. For example, the ZymoMS_Thermo protocol detected 0.005% (*M. smithii*, 28 reads), 0.095% (*S. enterica*, 483 reads), 0.007% (*E. faecalis*, 40 reads), and 0.021% (*C. perfringens*, 110 reads), while other extraction methods yielded comparable but slightly lower detection rates. The ZymoMS_Modified protocol identified 0.0004% (2 reads), 0.005% (21), 0.001% (4), and 0.004% (20) reads, while the ZymoMS_Promega protocol yielded 0.001% (4), 0.012% (56), 0.001% (4), and 0.004% (20) for the same taxa. These findings indicate that Illumina-based 16S rRNA gene amplicon sequencing exhibits limited sensitivity for taxa with very low relative abundances (< 0.5%) in complex microbial communities.

### Full-length 16S rRNA gene amplicon sequencing on the ONT platform

**Figure 2:**
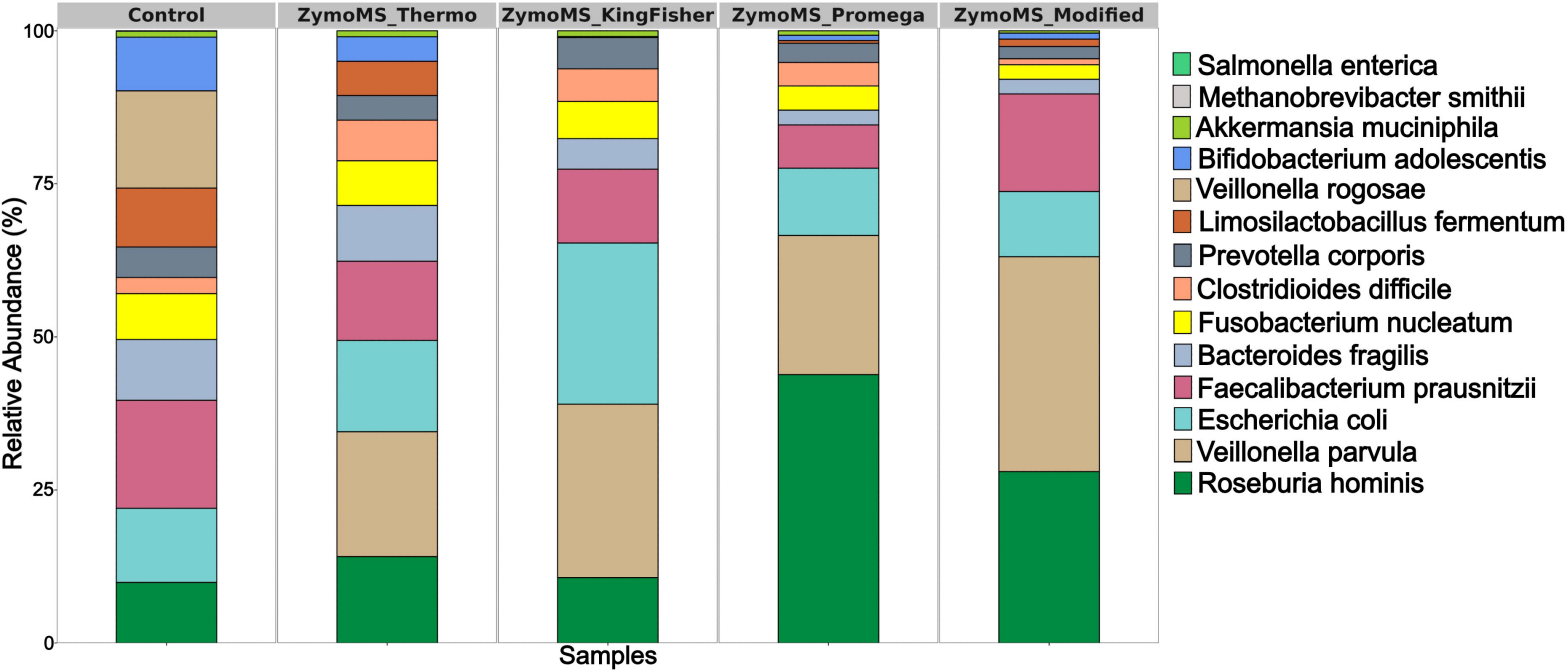
Relative abundances of bacterial species identified based on full-length 16S rRNA gene amplicon sequencing on the ONT platform. Bar plots show the relative abundances (%) of bacterial species detected across four DNA extraction methods in comparison with the ZymoBIOMICS Gut Microbiome Standard (Сontrol). All detected taxa are shown, however, species with relative abundances below 0.5% are difficult to distinguish due to their low representation. Full-length 16S rRNA gene sequencing on the ONT platform improved species-level resolution for dominant taxa, while the presence of low-abundance species was confirmed by BLAST alignment. Detailed results are provided in **Supplementary Table S3**.

The number of reads obtained from 16S rRNA gene amplicon sequencing on the ONT platform is summarized in **Supplementary Table S1**. Of the 18 bacterial species included in the ZymoBIOMICS Gut Microbiome Standard, 11 were identified. Eukaryotic organisms (*Candida albicans* and *Saccharomyces cerevisiae*) were not detected, as they lack 16S rRNA genes. Similarly, low-abundance microorganisms (< 0.5%) such as *M. smithii, S. enterica, E. faecalis*, and *C. perfringens* were also not detected using the Emu pipeline (**Figure 2**). However, BLAST alignment of ONT reads to the reference genomes of these minor species confirmed their presence at low frequencies in several extraction protocols. For *S. enterica*, 0.078% (63 reads) were detected in ZymoMS_KingFisher, 0.025% (14) in ZymoMS_Promega, 0.106% (203) in ZymoMS_Thermo, and 0.039% (21) in ZymoMS_Modified. For *E. faecalis*, 0.005% (4 reads) were detected in ZymoMS_KingFisher, 0.007% (4) in ZymoMS_Promega, 0.027% (52) in ZymoMS_Thermo, and 0.022% (12) in ZymoMS_Modified. Similarly, *C. perfringens* was identified with 0.018% (10 reads) in ZymoMS_Promega and 0.058% (110) in ZymoMS_Thermo. Gram-positive bacteria, including *Bifidobacterium adolescentis* and *Limosilactobacillus fermentum*, were extracted more efficiently using the ZymoMS_Thermo protocol, likely due to the bead-beating step that facilitating cell wall disruption.

Analysis of full-length 16S rRNA gene amplicon sequencing data obtained on the ONT platform revealed the presence of the major bacterial species included in the ZymoBIOMICS Gut Microbiome Standard, except for *Veillonella rogosae*. This species was misidentified as *V. parvula* across all four extraction methods. The misidentification is attributed to the high sequence similarity between the 16S rRNA genes of these species, which share 98% homology (Arif et al., 2008). However, their whole genome shares only 67% homology, illustrating the limitations of 16S rRNA gene amplicon sequencing in distinguishing closely related taxa. These finding highlights the importance of complementing amplicon-based approaches with MS to achieve higher species-level accuracy. Among the tested extraction methods, the microbial composition derived from the ZymoMS_Thermo protocol most closely matched the theoretical composition of the ZymoBIOMICS Gut Microbiome Standard (**Supplementary Table S3**).

### Metagenome sequencing for comprehensive microbial profiling

**Figure 3:**
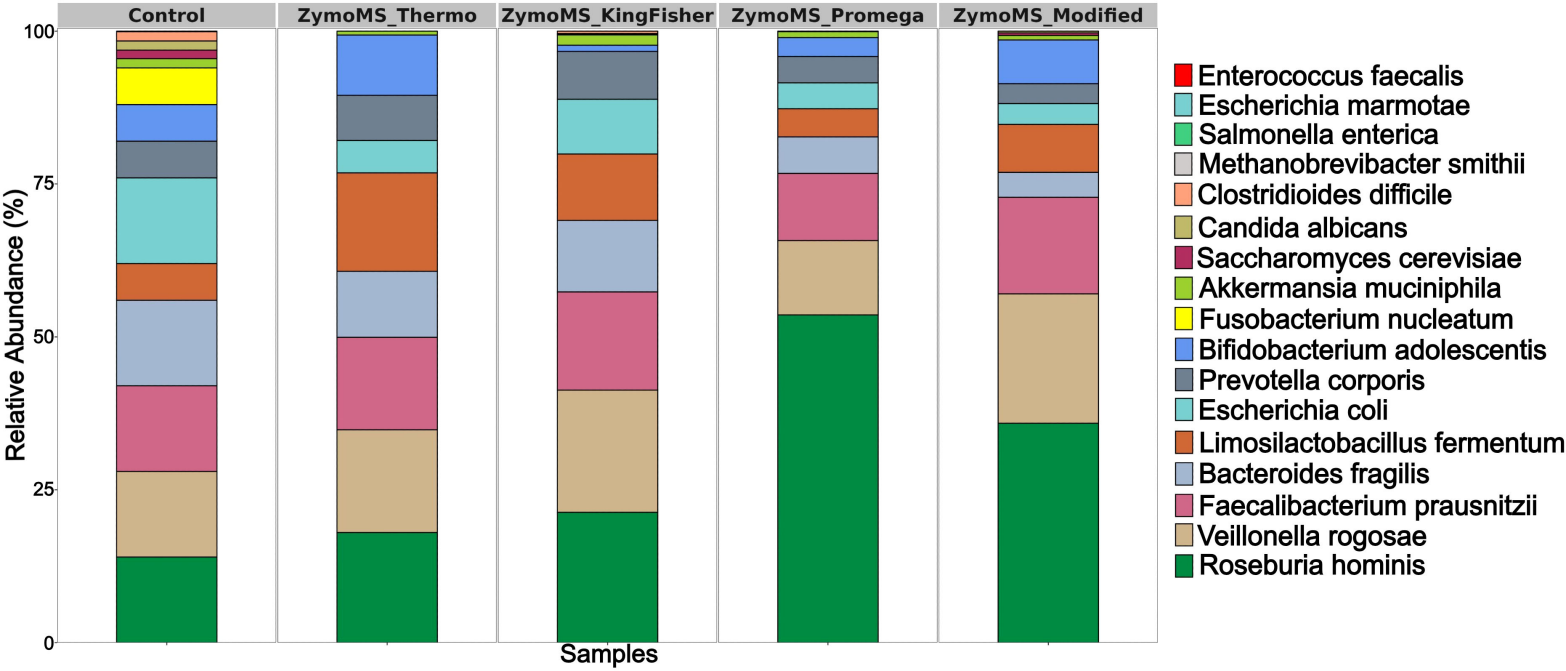
Relative abundance (%) of bacteria at the species level identified by MS on the Illumina platform.

The number of reads obtained from MS on the Illumina platform is shown in **Supplementary Table S1**. The observed difference in read counts between samples extracted using the ZymoMS_Promega and ZymoMS_Modified protocols can be attributed to the substantially longer average read length in the latter (10,142 bp vs. 1,327 bp), resulting in lower number of reads but longer fragments (**Table 1**). MS on the Illumina platform detected all major bacterial species present in the ZymoBIOMICS Gut Microbiome Standard, while species with theoretical abundance below 0.5% mostly remained undetected. A notable exception was *M. smithii*, which was successfully identified in the ZymoMS_KingFisher and ZymoMS_Modified samples (**Figure 3**, **Supplementary Table S4**).

Moreover, some reads obtained with the ZymoMS_KingFisher protocol were assigned to *Escherichia marmotae* (0.002%), a bacterial species not included in the ZymoBIOMICS Gut Microbiome Standard (**Supplementary Table S4**). This low-level false-positive assignment likely resulted from a bioinformatic classification artifact. Notably, *V. rogosae* was correctly identified using MS, in contrast to 16S rRNA gene amplicon sequencing, which misidentified it as *V. parvula*. These results underscore the superior accuracy of MS in identifying major bacterial species and highlight its value for accurate and comprehensive microbiome profiling.

Among the tested methods, the custom ZymoMS_Modified protocol for HMW DNA extraction provided the most comprehensive representation, achieving a complete match with the theoretical composition of the ZymoBIOMICS Gut Microbiome Standard. In contrast, while the ZymoMS_Thermo protocol yielded a bacterial composition similar to the control, it failed to detect the two eukaryotic species, *C. albicans* and *S. cerevisiae*. This was likely because the lysis conditions optimized for bacterial cells in the ZymoMS_Thermo were insufficient to disrupt the more robust fungal cell walls. Consequently, the ZymoMS_Modified protocol, with its more rigorous and extended lysis steps, proved superior for the simultaneous extraction of DNA from both bacterial and eukaryotic members of a microbial community. Furthermore, both the ZymoMS_Thermo and ZymoMS_Modified protocols demonstrated enhanced efficiency in extracting nucleic acids from Gram-positive bacteria (*B. adolescentis* and *L. fermentum*), as evidenced by their higher relative abundances compared to other methods (**Supplementary Tables S3**, **S4**).

Although many bioinformatics tools allow strain-level identification (De Coster and Rademakers, 2023), no strains identical to those declared in the ZymoBIOMICS Gut Microbiome Standard were detected in strain-level analysis. BLAST alignments of reads to the reference strains included in the standard showed high sequence homology, confirming the accuracy of species-level identification (**Table 2**). Sequences obtained using the ZymoMS_KingFisher protocol exhibited 97.8% to 100% homology with NCBI references, while those from ZymoMS_Modified ranged from 96.2% to 99.9%. Reads from the ZymoMS_Promega and ZymoMS_Thermo protocols demonstrated 97.7% to 99.6% and 98.1% to 99.9% homology, respectively.

**Table 2:** Average percent identity between obtained reads and reference genomes (alignments > 95% identity).

Genome			Sample			
	Strain ID	GenBank	ZymoMS_ Thermo	ZymoMS_ KingFisher	ZymoMS_Promega	ZymoMS_Modified
*Faecalibacterium prausnitzii*	AP34BHI	GCA_028743395.1	98.9	98.7	98.7	98.8
*Veillonella rogosae*	AC2811 AN NA 2	GCA_028743475.1	99.6	99.6	99.5	99.5
*Roseburia hominis*	OB EAV1–11 DCM	GCA_028743455.1	98.1	97.8	97.7	97.7
*Bacteroides fragilis*	OB EAV1–11 D6 FAA	GCA_028743275.1	99.7	99.6	99.6	99.6
*Prevotella corporis*	OB21 FMU 4	GCA_028743775.1	99.2	99.1	99.1	99.1
*Bifidobacterium adolescentis*	LMG 10502	GCA_028743295.1	98.5	98.4	98.4	98.5
*Fusobacterium nucleatum*	2/1/50A	GCA_028743415.1	98.4	97.8	97.8	96.2
*Lactobacillus fermentum*	B-1840	GCA_030770375.1	99.3	99.4	99.4	99.4
*Clostridium difficille*	P4D3A1-1	GCA_028743315.1	99.1	99.3	99.3	99.4
*Akkermansia muciniphila*	OB21 FAA NB 28	GCA_028743255.1	99.7	99.2	99.0	99.3
*Methanobrevibacter smithii*	DSM 861	GCA_000016525.1	99.9	98.9	99.6	99.4
*Salmonella enterica*	B-4212	GCA_028743635.1	99.2	99.6	99.6	99.6
*Enterococcus faecalis*	IP101412 AER FAA 2	no number	not detected	100.0	99.2	99.9
*Clostridium perfringens*	OB21 TSA 19	GCA_028743735.1	not detected	100.0	not detected	not detected
*Escherichia coli*	JM109	GCF_009916115.1	99.1	99.4	99.4	99.4
*Escherichia coli*	B-3008	GCA_028743355.1	99.6	98.8	99.1	98.9
*Escherichia coli*	B-2207	GCA_028743335.1	98.9	98.9	98.9	98.9
*Escherichia coli*	B-766	GCA_028743755.1	99.7	99.6	99.6	99.6
*Escherichia coli*	B-1109	GCA_028743555.1	98.3	99.1	99.2	99.1
*Candida albicans*	IHEM 3108	no number	not detected	not detected	not detected	not detected
*Saccharomyces cerevisiae*	Y-567	GCA_030867715.1	not detected	not detected	not detected	not detected

The high alignment rates to bacterial strains in the ZymoBIOMICS Gut Microbiome Standard underscore the utility of MS for comprehensive and accurate microbiome profiling, while emphasizing the importance of optimized extraction protocols to enhance the detection of low-abundance taxa.

**Figure 4:**
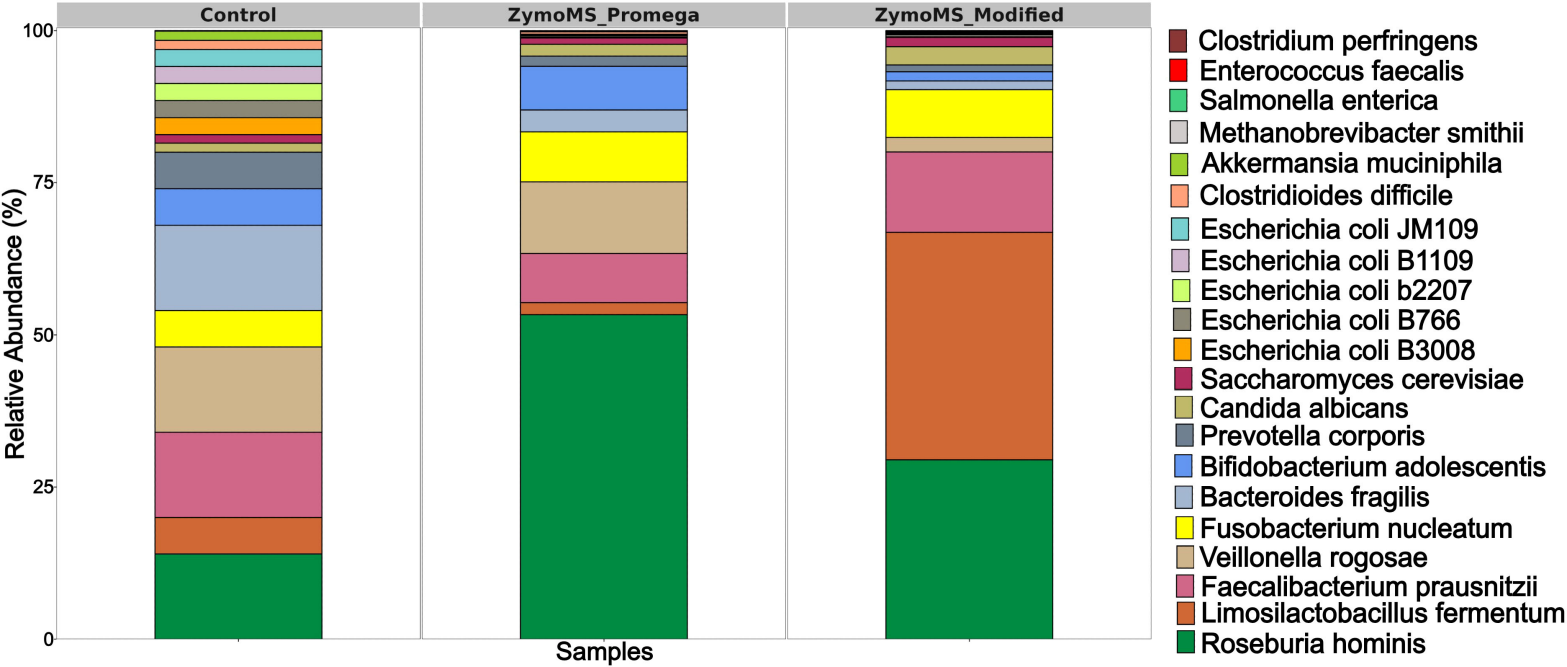
Relative abundance (%) of bacteria at the species level obtained with MS on the ONT platform.

The ZymoMS_Promega and ZymoMS_Modified protocols were selected for sequencing on the ONT platform because of their ability to recover HMW DNA. Analysis of the sequencing data using the Emu pipeline did not detect the low-abundance bacterial species present in the standard. To confirm their presence, a direct read mapping approach was employed against the corresponding reference genomes. This sensitive alignment-based analysis successfully identified reads aligning to all bacterial species included in the ZymoBIOMICS Gut Microbiome Standard (**Figure 4**, **Supplementary Table S5**), confirming the presence of their DNA in the sequencing libraries. The primary challenge, therefore, lies not in the wet-lab protocol but in the bioinformatic detection limits for very low-abundance taxa.

Notably, the quality of reads mapped to minor bacteria was, for the most part, significantly lower than that of reads mapped to dominant taxa (**Supplementary Tables S6** and **S7**), likely reflecting their initially low abundance in the sample. Detecting low-abundance bacteria remains challenging with current sequencing technologies. A higher sequencing depth and greater number of contiguous genome fragments were interpreted as indicators of superior DNA integrity and extraction efficiency, whereas lower coverage and increased genome fragmentation suggested degraded or incomplete DNA recovery. A statistical comparison of the coverage depth between the two extraction kits revealed a significant difference (Wilcoxon rank sum test, p-value < 0.05), supporting the conclusion that the choice of extraction method significantly influences DNA yield and quality (**Supplementary Figure S1**).

## Discussion

Studies utilizing ZymoBIOMICS Gut Microbiome Standard have demonstrated its effectiveness in benchmarking DNA extraction protocols, particularly in assessing their ability to recover diverse microbial communities without bias. Comparative analyses using the ZymoBIOMICS Gut Microbiome Standard have shown that mechanical lysis methods often introduce fragmentation, while enzymatic approaches preserve DNA integrity for long-read sequencing. The frequent use of this standard across various platforms underscores its critical role in addressing technical variability and advancing microbiome research (Bertolo et al., 2024; Lin et al., 2024).

Our findings confirmed that mechanical lysis methods, such as those employed in the MagicPure^®^ and PureLink^™^ kits, lead to significant fragmentation. In contrast, enzymatic methods, including those in the Wizard^®^ kit and the custom protocol, successfully preserved HMW DNA suitable for MS on the ONT platform. The ability to preserve DNA integrity is crucial for applications requiring long reads, such as species-level identification or strain-level analysis. The primary strength of using the ZymoBIOMICS Gut Microbiome Standard, as demonstrated in this study, is the ability to make definitive, quantitative assessments of DNA extraction and sequencing protocols against a known ground truth. This approach unequivocally identified the performance characteristics of each method. To complement these controlled findings, we also sought to verify the practical utility of the top-performing protocols. In a parallel study using complex, biological mouse gut microbiota samples, the ZymoMS_Thermo and ZymoMS_Modified protocols were applied and yielded high-quality sequencing data (Strokach et al., 2025). The successful application of these methods in a real-world scenario demonstrates their robustness for DNA recovery from complex biological matrices, supporting their broader applicability beyond synthetic communities.

Despite these advances, both Illumina and ONT sequencing approaches demonstrated limitations in identifying taxa with abundances below 0.5%, which remains a common challenge in microbiome research. Low-abundance species are often overlooked due to insufficient sequencing depth, extraction or algorithmic constraints. Increasing sequencing coverage can improve the detection of rare taxa, but this inevitably raises costs and may limit the feasibility of large-scale studies. A recent study systematically evaluated the influence of sequencing depth on microbial diversity estimates (Veselovsky et al., 2025) and demonstrated that increasing the number of reads per sample improved taxonomic resolution up to a certain threshold, beyond which additional sequencing provided minimal benefit. Taxa with relative abundances below 0.5% should be interpreted with caution.

Recent advances in nanopore sequencing technologies have significantly enhanced the accuracy and taxonomic resolution of full-length 16S rRNA gene amplicon analyses. Parameters that can influence the detection of bacterial species, particularly those present in low abundance, include basecalling mode, sequencing depth, read quality, and bioinformatic post-processing. The choice of basecalling mode in ONT workflows - specifically the high-accuracy “hac” and super-accuracy “sup” modes - can have measurable effects on per-base accuracy and, consequently, on the reliable detection of rare taxa. Methodological developments have demonstrated that ONT’s R10.4.1 chemistry, combined with the latest basecallers, can achieve Q20 and even Q28 accuracy levels (Zhang et al., 2023, 2024), narrowing the performance gap between nanopore and short-read platforms such as Illumina. This improvement may be particularly beneficial for distinguishing low-abundance or closely related bacterial species that were previously unresolved due to read-level errors. Nevertheless, evidence from recent comparative studies indicates that these improvements in basecalling accuracy may not always translate into substantially different microbiome diversity outcomes. For instance, (Aja-Macaya et al., 2025) reported that although the “sup” mode provided higher per-base accuracy, the overall microbial diversity and taxonomic profiles were largely consistent with those obtained using the “hac” mode. Similarly, benchmarking analyses using the ZymoBIOMICS Gut Microbiome Standard (Veselovsky et al., 2025) demonstrated that both “hac” and “sup” basecallers yielded highly comparable read counts, precision, recall, and F1-scores, with negligible impact on taxonomic assignment accuracy. The primary distinction was the increased computational demand associated with sup mode.

Despite these observations, the detection of underrepresented bacterial species remains a persistent challenge, particularly when their relative abundance falls below 0.5%. To improve the identification of such taxa, several complementary strategies can be employed. Increasing sequencing depth remains the most direct approach, as it enhances the likelihood of capturing rare microbial signatures, though this must be balanced against cost and diminishing returns beyond a certain threshold. Another promising approach involves adaptive sampling, where real-time nanopore sequencing selectively enriches for underrepresented taxa or specific genomic regions. On the computational side, the use of error-correction tools, such as Medaka or NanoPolish, combined with high-quality reference databases (e.g., SILVA, GTDB), can improve classification accuracy by reducing base-level noise. Additionally, hybrid assembly or co-binning strategies that integrate ONT long reads with Illumina short reads can increase confidence in low-abundance species identification and recover more complete genomes. Future research should also explore machine-learning–based taxonomic classifiers trained on error-aware nanopore data, which could better handle sequencing artifacts and improve the detection of rare taxa in complex communities. Collectively, these methodological and computational refinements are expected to advance the ability to profile low-abundance microbial members, enabling more accurate characterization of microbial communities, detection of potential biomarkers, and understanding of subtle ecological dynamics in clinical, environmental, and industrial microbiomes.

In our study, MS on Illumina proved to be the most comprehensive approach, accurately detecting all declared bacterial species in the ZymoBIOMICS Gut Microbiome Standard, including *V. rogosae*, which was misidentified as *V. parvula* in 16S rRNA gene amplicon sequencing data. In contrast, 16S rRNA gene amplicon sequencing is more suitable for qualitative analysis due to its biases in the quantitative determination of bacterial composition in the ZymoBIOMICS Gut Microbiome Standard. For instance, it has been previously shown that the V3–V4 region of 16S rRNA gene of *Bacillus cereus* is identical to that of *B. mobilis*, making these species indistinguishable through this approach. Similarly, the V3–V4 region of 16S rRNA gene of *Streptococcus pneumoniae* differs by only a single nucleotide from that of *S. infantis* (Gwak and Rho, 2020). These limitations underscore the need for improved algorithms capable of handling sequencing errors and distinguishing closely related species, particularly when sequencing the complete 16S rRNA gene.

We note that the DADA2 (Callahan et al., 2016) pipeline provides high accuracy for short-read Illumina data, but in many cases its performance is limited to genus-level resolution due to the short amplicon size, and it may misclassify closely related species. Importantly, several studies have demonstrated that DADA2 is not well-suited for ONT data because it was originally developed for the low error rates characteristic of Illumina sequencing. When applied to ONT reads, DADA2 performs poorly due to its stringent error model assumptions and inability to handle the higher indel and substitution error rates inherent to nanopore sequencing (Callahan et al., 2019; Santos et al., 2020; Chang et al., 2024). These reports consistently indicate that DADA2 tends to over-split true biological variants and produce unreliable ASV inference when used on ONT datasets.

In contrast, the Emu (Curry et al., 2022) pipeline is designed for full-length 16S rRNA gene amplicon reads generated by ONT, allowing for species-level classification; however, it exhibits reduced sensitivity for low-abundance taxa and is highly dependent on the completeness of the reference database. We also emphasize that both tools may contribute to discrepancies in the detection of rare or closely related species. Recent comparative studies further highlight these differences, showing that taxonomic resolution and sensitivity can vary substantially depending on the chosen pipeline (Strokach et al., 2025; Veselovsky et al., 2025).

Challenges in analyzing 16S rRNA gene amplicon sequencing data on the ONT platform may stem from multiple factors. These include imperfections in primer complementarity (Frank et al., 2008), periodic taxonomic restructuring in reference databases (Vanhee et al., 2024), and the inherent detection limits of the ONT 16S rRNA gene amplicon sequencing platform (Lao et al., 2023). These factors collectively highlight the need for continuous improvement in both laboratory protocols and computational pipelines to achieve more accurate and reliable microbiome analyses. The ZymoBIOMICS Gut Microbiome Standard was used to quality control the microbiome studies. The taxonomic analysis results showed that current bioinformatics methods are insufficiently accurate in identifying taxa with a relative abundance of less than 0.5% (Veselovsky et al., 2025).

When analyzing the ZymoMS_Thermo sample using two major sequencing strategies – 16S rRNA gene amplicon sequencing and MS – we observed a comparable microbial composition, despite using different Illumina platforms for each. This indicates that MS offers robust performance in profiling well-defined microbial communities, even across distinct sequencing setups. However, for 16S rRNA-based taxonomic profiling, ONT outperformed Illumina in terms of species-level resolution, owing to its ability to sequence the full-length 16S rRNA gene amplicon. These findings support the view that the choice of sequencing platform and method should be aligned with specific research goals. Moreover, previous studies have shown that using a hybrid assembly approach combining Illumina and ONT reads can further improve the resolution and number of identified contigs (Overholt et al., 2020).

The variability in DNA extraction efficiency across methods further influenced the outcomes of sequencing. Protocols like ZymoMS_Thermo and the custom method demonstrated better performance in extracting nucleic acids from Gram-positive bacteria, underscoring the critical role of mechanical lysis, such as bead-beating, in recovering DNA from resilient cell types.

## Conclusions

The accuracy of 16S rRNA gene amplicon sequencing can be enhanced by selecting appropriate DNA extraction methods. Using a combination of the PureLink™ Microbiome DNA Purification Kit (Thermo Fisher Scientific, USA) for DNA extraction and 16S rRNA gene amplicon sequencing on the ONT and Illumina platforms, we obtained results that closely matched the theoretical composition of the ZymoBIOMICS Gut Microbiome Standard. The Wizard^®^ Genomic DNA Purification Kit (Promega) and the custom protocol proved most suitable for MS on the ONT platform, owing to their ability to recover HMW DNA. The PureLink™ kit and the custom protocol also demonstrated high efficiency in recovering DNA from Gram-positive bacteria.

Analysis of 16S rRNA gene amplicon sequencing data may lead to the misclassification of closely related species due to the high sequence homology of the 16S rRNA gene. This limitation can be addressed by MS, which provides greater taxonomic resolution. However, taxa with low-relative abundance (< 0.5%) remain difficult to detect with both 16S rRNA amplicon sequencing and MS approaches, primarily due to methodological constraints and limited sequencing depth. Despite these limitations, 16S rRNA gene amplicon sequencing remains a cost-effective tool for large-scale studies aimed at broad microbial community profiling. For studies requiring higher taxonomic resolution and comprehensive characterization, MS is preferable.

## Data Availability

The datasets presented in this study can be found in online repositories. The names of the repository/repositories and accession number(s) can be found in the article/**Supplementary Material**.
