# Supplementary material for: Impact of DNA extraction techniques and sequencing approaches on microbial community profiling accuracy
Source: Front Microbiomes. 2025 Dec 16;4:1688681. doi: 10.3389/frmbi.2025.1688681 (PMC12993687; doi:10.3389/frmbi.2025.1688681)
Supplement: Supplementary file 1 [file DataSheet1.zip › Supplementary/Captions.docx]

**Table S1.** Total reads obtained with different DNA extraction methods.

**Table S2.** Alpha diversity indices of bacterial communities obtained with different sequence methods.

**Table S3.** Taxonomic distribution of species obtained with metagenome sequencing on Illumina.

**Table S4.** Number of reads and theoretical composition of bacterial species identified with different DNA extraction and sequencing methods.

**Table S5.** Number of reads mapped to reference genomes from ZymoBIOMICS Gut Microbiome Standard for metagenome sequencing on ONT.

**Table S6.** Mapping quality and number of reads for ZymoMS_Promega (ONT)

**Table S7.** Mapping quality and number of reads for ZymoMS_Modified (ONT)

**Figure S1.** Comparison of the coverage depth between ZymoMS_Promega and ZymoMS_Modified
