# Supplementary figures and images for: Impact of DNA extraction techniques and sequencing approaches on microbial community profiling accuracy
Source: Front Microbiomes. 2025 Dec 16;4:1688681. doi: 10.3389/frmbi.2025.1688681 (PMC12993687; doi:10.3389/frmbi.2025.1688681)

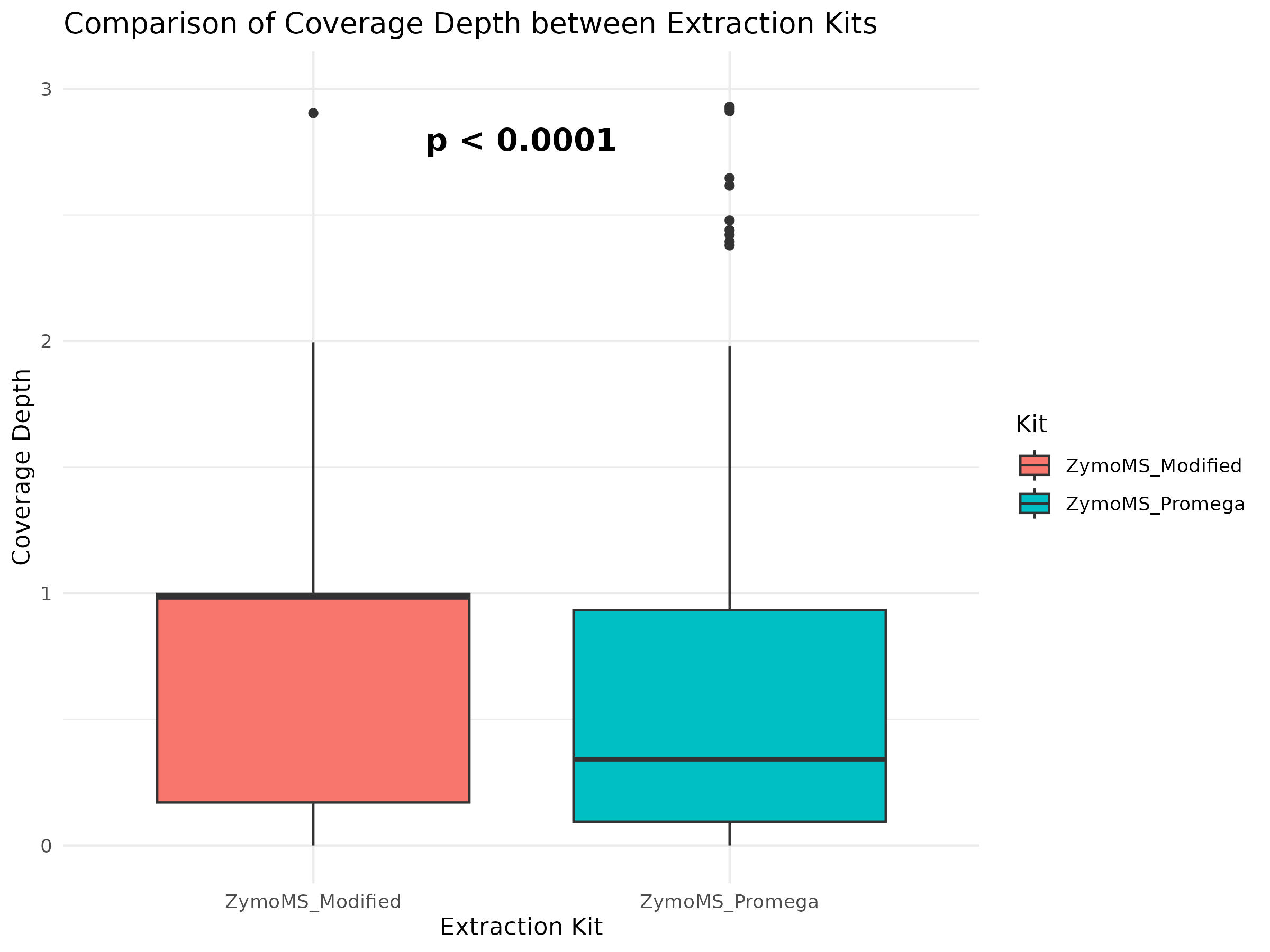

Supplement: Supplementary file 1 [file DataSheet1.zip › Supplementary/Figure S1.jpeg]
